# Investigating the Overlap of *Hikikomori* and Autism Spectrum Disorder: A Case Report

**DOI:** 10.3390/medicina61040637

**Published:** 2025-03-31

**Authors:** Marianna Moro, Alessia de Gioia, Giulia D’Amario, Valentina Napoli, Ilaria Venezia, Federica Mirra, Martina D’Ambrosio, Romina Venditti, Simona Sestito, Andrea De Stefano, Sara Di Domenico, Domenico Romeo, Eugenio Mercuri, Claudia Brogna

**Affiliations:** 1 Pediatric Neurology Unit, Università Cattolica del Sacro Cuore, 00168 Rome, Italy; marianna.moro@guestpoliclinicogemelli.it (M.M.); alessia.degioia01@icatt.it (A.d.G.); giuliadamario@gmail.com (G.D.); valenap86@gmail.com (V.N.); fedemirra20@gmail.com (F.M.); martina.dambrosio01@icatt.it (M.D.); romina.venditti01@icatt.it (R.V.); simona.2011@libero.it (S.S.); andrea.destefano.1996@gmail.com (A.D.S.); domenicomarco.romeo@policlinicogemelli.it (D.R.); eugeniomaria.mercuri@policlinicogemelli.it (E.M.); 2 Psychologist and Psychoterapist, Piazza Attilio Omodei Zorini 11, 00166 Rome, Italy; sara.didomenico@yahoo.it; 3 Pediatric Neurology Unit, Fondazione Policlinico Universitario “A. Gemelli”, IRCCS, 00168 Rome, Italy

**Keywords:** *hikikomori*, Autism Spectrum Disorders, case study, social isolation

## Abstract

*Hikikomori* is a form of social withdrawal lasting more than 6 months with significant associated functional impairment. To date, numerous studies confirm the presence of this condition not only in Japan, where it was first described, but also globally abroad. This is an underestimated clinical condition, and it is emerging especially in adolescents and young adults, representing an increasing management problem for families and society. Prevalence ranges from 1.1% to 6.7%. *Hikikomori* can be associated with other neurodevelopmental disorders, such as Autism Spectrum Disorder (ASD). Indeed, ASD and *hikikomori* share numerous characteristics confirmed by functional neuroimaging studies that have highlighted in both conditions the presence of alterations in cerebral regions related to social functioning. We present a case report regarding the history of a 14-year-old girl with characteristics compatible with ASD and *hikikomori*. At present, there are no specific treatments approved for *hikikomori* in ASD patients. Further studies are necessary to understand the link between the two conditions, the boundary, and possible overlap.

## 1. Introduction

The term *hikikomori* derives from the verb “hiki”, meaning to retreat, and “komoru”, meaning to seclude oneself. *Hikikomori* is characterized by a pathological form of social withdrawal or isolation lasting more than six months accompanied by significant functional impairment or distress associated with this isolation [1]. *Hikikomori* cases have been reported not only in Japan, where this condition was initially identified, but also globally across several countries, including Australia, the USA, Spain, Bangladesh, India, Iran, Korea, Taiwan, Thailand, and Oman [2,3], so much so that some authors have described it as a “culture-bound syndrome” [4]. So far, the prevalence of this condition is not yet well-recognized, representing an increasing management problem for families and society. Age at onset is typically during adolescence or early adulthood. However, onset after the third decade is not rare [4]. Hikikomori has been included as an example of cultural concepts of distress in the Diagnostic and Statistical Manual of Mental Disorders—Fifth Edition TR, even if some authors wonder whether this choice was premature or justified by actual scientific knowledge [5]. Social isolation and the avoidance of social contexts, such as school, workplaces, and interpersonal relations, are the main features of hikikomori syndrome. According to the guidelines for Evaluation and Support of Hikikomori given by the MHLW (Ministry of Health, Labour, and Welfare) of the Japanese Government, the diagnostic criteria for *hikikomori* include [6] (a) pronounced social isolation with a refusal to leave the house; (b) lack of interest in ordinary activity, including school or the workplace; (c) duration of symptoms of at least 6 months; (d) absent or partial fulfillment of the diagnostic criteria for schizophrenia, intellectual disability, or other mental disorders; and (e) exclusion from this diagnostic category of individuals who still maintain interpersonal relations (e.g., with friends), although they do not attend a school or the workplace. However, considering the complexity of this disorder, new diagnostic criteria of *hikikomori* for future DSM/ICD diagnostic systems have been proposed to also include those conditions that are often excluded from this diagnosis [7] while also taking into account a grading of severity of the condition. Individuals who occasionally leave their home (2–3 days/week), rarely leave their home (1 day/week or less), and rarely leave a single room may be characterized as having mild, moderate, and severe hikikomori, respectively. A pre-*hikikomori* condition has been also suggested, which refers to individuals with a duration of continuous social withdrawal of at least 3 (but not 6) months. Furthermore, Kato et al. [7] also introduced some necessary specifiers of this condition (including lack of social participation; lack of in-person social interaction; indirect communication; loneliness; a co-occurring condition; age at onset; family pattern and dynamics; cultural background; and intervention).

The etiology of *hikikomori* remains unclear, although numerous studies have proposed various hypotheses involving societal influences and family factors, such as a parenting style characterized by an overprotective approach typical of Japanese parents, most known as “amae”. Also, socioeconomic status and biological or biochemical factors, including specific blood biomarkers, have been considered [8,9].

*Hikikomori* has been found to be associated with various psychiatric disorders, such as anxiety disorders and depression, as well as Autism Spectrum Disorder (ASD). Indeed, a classification of two types of *hikikomori* has been proposed: primary *hikikomori*, which occurs in the absence of other psychiatric comorbidities, and secondary *hikikomori*, where social withdrawal can be attributed to an underlying psychiatric disorder [8]. *Hikikomori* and high-functioning ASD share several features, including social anxiety, challenges in social communication (both verbal and non-verbal), and isolation [10]. It is necessary to specify, however, that patients with ASD exhibit symptoms early in the first years of life (e.g., difficulties with social interaction, gaze modulation, and voice intonation, absence or scarcity of symbolic gestures, etc.), which are generally absent in individuals with primary *hikikomori* during childhood, as onset occurs later. The hypothesis that autistic traits in individuals with *hikikomori* could be considered a particular presentation of ASD has just been postulated, mainly in adult patients [11,12,13]. High-functioning individuals with ASD are more at risk of developing Internet addiction and Internet gaming disorder (IGD), which would further reduce social interaction, as is typical of *hikikomori.* The coexistence of ASD and *hikikomori* is often indicated by early onset of symptoms (in early childhood), followed, during the adolescence, by social withdrawal that appears more severe than expected given the patient’s cognitive level and adaptive functioning, leading to a significant distress for the patient. Generally, this distress does not occur in individuals with only ASD, who, on the contrary, tend to prefer isolation. So far, in the literature, there are several clinical studies regarding *hikikomori* syndrome in the adult population, and only a few studies on adolescents and young adults have been reported (see Table 1 for details). Furthermore. only a few studies have explored the comorbidity between ASD and *hikikomori* (see Table 2 for details) [13,14,15,16,17].

We present the case of a young girl presenting with *hikikomori syndrome* associated with clinical aspects and a clinical history compatible with high-functioning Autism Spectrum Disorder.

Written consent from the caregiver was obtained. The study was approved by the ethics committee of our institution (ID: 3418; prot. N. 0037324/20).

## 2. Case Report

A 14-year-old girl was admitted to the Neuropsychiatric unit of Fondazione Policlinico Gemelli IRCCS in Rome, where she was referred by her parent due to symptoms related to anxiety and school refusal. She was born in Texas, where she lived for seven years before moving to Italy. Her father had a diagnosis of depression, treated with antidepressants, and a history of alcohol abuse. Her family history revealed a paternal uncle with an alcoholic and depression diagnosis and a cousin with Autism Spectrum Disorder. The girl was born at term without complications during pregnancy or delivery, with an appropriate birth weight. She achieved motor milestones at the expected times. However, since kindergarten, her mother noted social isolation, solitary play, and selective friendships, which was confirmed by the teachers. Stereotyped movements and restricted and repetitive interests have been reported. The difficulties in the relationships persisted during primary school in Italy: the mother reported that the girl exhibited poor adaptability in social contexts, difficulties understanding irony and facial expressions, and struggles with making friends, often being bullied at school. Since 2019, she presented as progressively withdrawn from school and social activities, which was associated with insomnia characterized by difficulty falling asleep and a significant reduction in total sleep hours. She spent most of her time at home on her personal computer or engaged in reading and drawing Japanese manga. Her mother reported excessive use of screen time, which worsened during the COVID-19 pandemic.

She first came to our attention at the age of 14 years, following concerns raised by her teachers regarding numerous absences at school in the last year and difficulties in peer interactions and socialization. The school refusal started to appear 9 months before, and the girl progressively reduced her outings and closeness to the few selective friends she had, spending most of her time alone and locked in her room in front of video games or reading manga (“anime”).

After an initial outpatient visit, further diagnostic evaluations were made, including both cognitive and learning assessment. Her cognitive not verbal functioning evaluated by using Raven’s Matrices was in the normal range (her intelligence quotient was 112), while learning assessments confirmed difficulties in reading and writing.

Given the presence of a clinical history and symptoms resembling a picture typical of ASD, the patient underwent an Observation Schedule—Second Edition (ADOS-2) Module 3 evaluation, which was positive for ASD with a total score of 10, consistent with ASD, with a comparison score of 6, suggesting a moderate level of severity. Her greatest challenges were in verbal and non-verbal communication (gestures, eye contact, facial expressions), and her social engagement was somewhat atypical, marked by significant relational inhibition. Reciprocal social communication was limited in quantity, and the overall quality of the relationship was comfortable but unsupported by the patient. She exhibited a strong interest in art, particularly video games, spending most of her time on electronic devices, sometimes for the entire day and night. The Autism Diagnostic Interview—Revised questionnaire (ADI-R) administered to her mother indicated the presence of symptoms consistent with autism since the girl’s early childhood. Her mother reported an absence of typical gestures and difficulties in all forms of communication, both verbal and non-verbal, since childhood, restricted interests (drawing, reading books and manga), and stereotyped movements (in the upper limbs). Her primary concerns about her daughter centered on her social isolation and excessive time spent playing video games or reading manga at home.

During clinical evaluation, the patient exhibited mood flattening, attentional lability, difficulties in emotional processing, and poor self-esteem, associated with poor interest in social interactions and social anxiety. She reported non-suicidal self-injurious thoughts, without intent or planning. Her attention abilities revealed deficiencies in auditory attention, along with a learning disorder in reading, specifically in reading and writing speed. She was also assessed using the Kiddie-Schedule for Affective Disorders and Schizophrenia (K-sads), which revealed the presence of depressive disorder, characterized by persistent depressed mood, related to school anxiety, irritability, anger, and non-suicidal self-injury behavior. In the domain of anxiety, she reported fear of social situations, particularly being questioned in class, using public restrooms, and changing clothes in front of others, leading to severe functional impairment for over six months. Additionally, self-reported questionnaires administered to the patient, her mother, and teachers highlighted difficulties in attention focusing, social withdrawal, isolation, and some obsessive tendencies towards order and precision.

In conclusion, the clinical picture, according to the clinical history, the timing of the onset of clinical signs, and the psychological assessments, was compatible with high-functioning ASD with social anxiety, depressive mood, and features compatible with *hikikomori* syndrome (for details, see Table 3).

After the initial assessment, considering the presence of social anxiety, depressive symptoms, difficulties in social adjustment, and pragmatic communication, the patient began speech therapy to enhance her language and social skills, as well as cognitive–behavioral therapy focused on anxiety management and social training. She was also started on Alprazolam 0.25 mg in the morning and Aripiprazole up to 5 mg/day. She reported mood stabilization; hence, the Aripiprazole dosage was increased to 7.5 mg/day with good efficacy. The patient experienced a reduction in anxiety symptoms and non-suicidal self-injurious thoughts following the initiation of treatments. However, her attitude did not change, and she continued to spend several hours during the day playing video games or reading manga at home.

## 3. Discussion

In the 2000s, Japanese society became aware of the social phenomenon of *hikikomori*, as 1.2% of individuals experienced “*hikikomori*” in their lifetime in Japan. However, this should be considered a global, silent epidemic [17,18,19,20,21,22,23,24].

These individuals believe that the only way to survive is by withdrawing from society and isolating themselves in their rooms [14,16]. So far, several studies have found that the majority of individuals with *hikikomori* also have an associated mental disorder [25,26,27]. Koyama et al. reported that 45.5% of individuals with *hikikomori* did not have a lifetime prevalence of another psychiatric disorder; however, personality disorders, psychotic disorders, and neurodevelopmental disorders were not considered in the screening process [15]. A recent review highlighted that psychiatric disorders and symptoms may trigger, co-occur with, or be consequential to *hikikomori*, with differences observed between the adolescents and the young adults. However, the studies mentioned in the literature are often heterogeneous regarding the samples of patients, the study protocols, etc. [28].

Therefore, this issue is often underestimated in society, especially among adolescents and young adults, and an accurate diagnosis of *hikikomori* condition is more complex. Several factors must be considered, including gender (the prevalence of *hikikomori* is four times higher in men than in women) [29], being a firstborn (due to the role of preserving the family), experiences of bullying, being from a middle-class family [30], and the family’s failure to intervene, which allows the condition to solidify.

There are few reports regarding the etiology of this condition. Hayakawa et al. investigated blood biomarkers of *hikikomori*, and *hikikomori* was associated with lower serum uric acid levels in men and lower high-density lipoprotein cholesterol levels in women compared to healthy controls [17].

While pharmacological treatment has to be considered when there are related psychiatric conditions, several non-pharmacological treatments for *hikikomori* should also be considered, such as jogging therapy, educational program for family members of *hikikomori*, music therapy based on cognitive–behavioral therapy, role-playing therapy using fictional narratives to promote empathy, animal-assisted therapy, and group therapy [19]. Wong et al. also suggested investing in youth mental health, such as providing more paid or unpaid job opportunities to keep young people engaged in society [31].

So far, there are only a few clinical cases reported on *hikikomori,* and most of them focus on adult patients. Maglia et al. described a case of an 18-year-old Italian boy diagnosed with *hikikomori* and video game addiction. Through systemic relational therapy, the patient was able to start his reintegration into the socio-family context, and then he regained interpersonal skills [14]. Katsuki et al., in their pilot case control study, suggested a link between *hikikomori* and autism spectrum conditions [17]. In particular, *hikikomori* sufferers are more likely to have autistic traits, and individuals with autism often have depressive symptoms compatible with *hikikomori*. This finding is supported by other studies [32].

Carpita et al. [11] described two cases of adult patients where the presence of underlying Autism Spectrum Disorder may have represented a sign of vulnerability for developing a full-blown case of hikikomori with IGD.

We described the case of a young girl affected by both high-functioning ASD and clinical features consistent with *hikikomori syndrome*. The diagnosis of ASD is a heterogeneous neurodevelopmental condition with varying degrees of symptom severity and with or without intellectual impairment. Its core symptoms include severe impairment in reciprocal and social communication and interactions, restricted interests, stereotyped behaviors, and impaired socio-emotional reciprocity and sensory integration processing. In high-functioning ASD subjects, the diagnosis is often delayed due to above-average intelligence levels and attempts to mask symptoms, preventing parents and teachers from suspecting differences from neurotypical individuals. In most cases, ASD diagnosis remains undiagnosed for years, coming to clinical attention only after the development of psychiatric comorbidities. ASD and *hikikomori* share some similar clinical manifestations related to the area of social skills, with a subtle difference; while social withdrawal is the most prevalent clinical sign in *hikikomori,* occurring at different ages, in ASD, there are social difficulties since childhood. In our case, the ASD diagnosis was suspected due to the presence of difficulties in social communication, impaired socio-emotional reciprocity, and selective interests that have been reported by the caregiver since an early age, as confirmed through the ADI-R interview. *Hikikomori* signs started to appear later during adolescence, which was confirmed by the presence of social withdrawal noticed by both the teachers and the family. Because of the overlapping of the two conditions, some authors consider *hikikomori* not a single clinical category with a specific psychopathology but rather a common phenotype with various underlying pathologies (for example, anxiety disorders, depression, etc.) [20]. For others, it is a stand-alone condition, diagnosed per se or as an exclusion diagnosis in the absence of symptoms meeting the diagnostic criteria for other conditions [21]. This clinical case confirmed that deficits in social interaction and social communication may be a vulnerability factor for developing *hikikomori* behaviors [13,33]. ASD and *hikikomori* do not only share some clinical aspects but also the same neuronal circuits in the brain. Functional MRI studies in *hikikomori* patients have highlighted structural and functional changes in several brain regions, including the prefrontal area, the temporal and parietal cortex, limbic structures (the hippocampus, the amygdala, and the insula), the striatum, and the cerebellum [34]. These regions belong to the so-called “social network” of the brain. Recent studies have highlighted the involvement of the same regions in ASD [35]. In particular, alterations affecting the temporal lobes and the posterior superior temporal sulcus (STS) have been widely studied and also associated with Autism Spectrum Disorders [36,37]. The superior temporal sulcus is the area where the “mirror neurons” system is located, and its function is deficient in individuals with ASD [38].

## 4. Conclusions

We reported the detailed history since childhood of an adolescent with high-functioning ASD who later developed *hikikomori*. So far, the presence of ASD in *hikikomori* has not been well-investigated, mainly in young populations. The co-presence of ASD and *hikikomori* is a possible finding in adolescents and young adults, and a diagnosis of high-functioning ASD must be taken into account in the presence of a clinical picture compatible with *hikikomori*. Further studies are needed to understand whether the two conditions overlap, like “two sides of the same coin”, sharing the same neuronal circuits and etiologies, or if they are two different entities coexisting.

At present, there are not yet sufficient specific treatments available approved for treating *hikikomori* condition in people with ASD. A recent case report suggested the use of a tele-operated robot to help these patients increase their sociability [39], but appropriate psychotherapeutic intervention and the use of specific psychiatric drugs also have to be considered in order to avoid possible psychiatric evolution.

Surely, further studies are needed to estimate the prevalence of *hikikomori* condition in the young autistic population, mainly in those with high-functioning symptomatology, in order to evaluate proper drug and non-drug treatment.

## Figures and Tables

**Table 1:** Reports in the literature regarding *hikikomori* in adolescents and young adults.

Authors and Years	Type of Manuscript	Demographics (Sex and Age)	Findings
Maglia et al., 2020 [14]	Case report	*n* = 1 (male) 18 years old	Systemic relational therapy as rehabilitation.
Koyama et al., 2010 [15]	Case (epidemiological) study	*n* = 1660 (male/female) 20–49 years old	There were 1.2% affected (1.8 male/0.4 female), while 45.5% did not have a lifetime prevalence of another psychiatric disorder.
Hayakawa et al., 2018 [16]	Case control study	*n* = 101 (male 46/female 55) 21 years old	Lower serum uric acid levels in men and lower high-density lipoprotein cholesterol levels in women with *hikikomori* compared with healthy controls.
Bellini B et al., 2024 [18]	Case control study	Hikikomori group *n* = 22 (11 males and 11 females) 12–17 years Control group *n* = 22 (8 males and 14 females) 11–17 years	*Hikikomori* syndrome is not secondary to a mood disorder, but prolonged social withdrawal can be a risk factor for depressive episodes. The *hikikomori* patients appeared less depressed, with a shorter duration of withdrawal, while their depressive symptoms may be intensified by longer social isolation.
Hamasaki et al., 2021 [19]	Case control study	Study group *n* = 20 (10 males/10 females); control group *n* = 88 (56 males/32 females) aged 12–15 years	The CBCL subscale “withdrawn” was found to be correlated to *hikikomori*. The independent variables correlated with *hikikomori* severity were “somatic complaints”, “anxious/depressed”, “overuse of the Internet”, and “lack of communication between parents”.
Hamasaki et al., 2022 [20]	Case control study	Study group *n* = 10 (7 males/3 females); control group *n* = 115 (58 males/57 females) aged 12–15 years	*Hikikomori* in Japan and France could be considered essentially the same phenomenon.
Silic, 2019 [21]	Case report	*n* = 1 male, 24 years old	The differential diagnosis of *hikikomori* with other conditions can be challenging. For the authors, *hikikomori* is a stand-alone clinical condition.
Benarous, 2022 [22]	Retrospective study	*n* = 191 adolescents (84 males/107 females) aged 12–18	Patients with *hikikomori* present distinct clinical features compared to those with other kinds of school refusal or social withdrawal.
Santona et al., 2023 [23]	Case study	*n* = 72 young adults (49 males/23 females) 12–33 years Mean age 22.5 years	An anxious attachment increased the odds of experiencing extremely high levels of interpersonal sensitivity and depression.

**Table 2:** Reports in the literature regarding *hikikomori* and ASD in adolescents and young adults.

Authors and Years	Type of Manuscript	Demographics (Sex and Age)	Findings
Carpita B et al., 2024 [11]	Case report	*n* = 2 (male) 40 and one 20-year-old	The study explores the correlation between autistic traits, mood symptoms, and traits of Internet gaming disorder.
Brosnan et al., 2023 [13]	Case (epidemiological) study	*n* = 646 (male 283/363 female) 16–24 years old	Psychological wellbeing and COVID-19 restrictions are associated with increased *hikikomori* risk in young adults, and both associations are mediated by higher levels of autistic traits.
Katsuki et al., 2020 [17]	Case control study	*n* = 324 (male 174/female 150) ≥15 years old (mean 31–35)	The study explores the association between autistic tendencies, difficulty in social communication, and social interaction.
Carpita et al., 2024 [12]	Population study	*n* = 1192 (577 male/615 female) university students	Autistic features can trigger the development of *hikikomori* in both sexes, which is in line with the rising hypothesis of a possible neurodevelopmental basis for different psychiatric disorders.
Yamada et al., 2023 [24]	Case control study	Control group *n* = 23 males with ASD; case group *n* = 16 males with ASD and hikikomori	ASD patients with *hikikomori* are more likely to have greater sensory abnormalities, comorbid atopic dermatitis, lower uric acid levels, and stronger depressive and anxiety tendencies.

**Table 3:** Neuropsychological assessments.

Neuropsychological Assessments		Scores
Autism symptomatology	Ados II Module 3	Social affection score: 9 Restricted and repetitive behaviors: 1 Total score: 10 Comparison score: 6
	ADI-R	Qualitative anomalies in the following: Area of mutual social interaction: 15 Area of communication: 10 Area of restricted, repetitive, and stereotyped patterns of behavior: 9
Neuropsychological profile	K-SADS	Depressive disorder area: depressed mood (irritability, anger, anhedonia, non-suicidal self-injury behavior) Anxiety area: social anxiety

## Data Availability

Data is contained within the article.
